# Microenvironment of ruptured cerebral aneurysms discovered using data driven analysis of gene expression

**DOI:** 10.1371/journal.pone.0220121

**Published:** 2019-07-22

**Authors:** Alexander P. Landry, Michael Balas, Julian Spears, Zsolt Zador

**Affiliations:** Division of Neurosurgery, Department of Surgery, St. Michael’s Hospital, Toronto, ON, Canada; College of Bioinformatics Science and Technology, CHINA

## Abstract

**Background:**

It is well known that ruptured intracranial aneurysms are associated with substantial morbidity and mortality, yet our understanding of the genetic mechanisms of rupture remains poor. We hypothesize that applying novel techniques to the genetic analysis of aneurysmal tissue will yield key rupture-associated mechanisms and novel drug candidates for the prevention of rupture.

**Methods:**

We applied weighted gene co-expression networks (WGCNA) and population-specific gene expression analysis (PSEA) to transcriptomic data from 33 ruptured and unruptured aneurysm domes. Mechanisms were annotated using Gene Ontology, and gene network/population-specific expression levels correlated with rupture state. We then used computational drug repurposing to identify plausible drug candidates for the prevention of aneurysm rupture.

**Results:**

Network analysis of bulk tissue identified multiple immune mechanisms to be associated with aneurysm rupture. Targeting these processes with computational drug repurposing revealed multiple candidates for preventing rupture including Btk inhibitors and modulators of hypoxia inducible factor. In the macrophage-specific analysis, we identify rupture-associated mechanisms MHCII antigen processing, cholesterol efflux, and keratan sulfate catabolism. These processes map well onto several of highly ranked drug candidates, providing further validation.

**Conclusions:**

Our results are the first to demonstrate population-specific expression levels and intracranial aneurysm rupture, and propose novel drug candidates based on network-based transcriptomics.

## Introduction

Saccular intracranial aneurysms are highly prevalent in the adult population (up to 5%) with rupture rates from 0.5% to over 20% per year[1]. The mechanisms leading to formation and rupture of intracranial aneurysms is not fully understood. The suggested process consists of chronic vascular remodelling paralleled by an inflammatory process which ultimately result in focal weakening and rupture of the arterial wall[2]. During this process, the extracellular matrix as well as cellular units of the arterial wall, endothelia and smooth muscle cells, become progressively disrupted paralleled by immune cells infiltration. The majority of this knowledge stems from histological studies of aneurysmal tissue[2] but our knowledge of the molecular processes occurring in each cellular unit remains incomplete.

Several medical strategies have been investigated to prevent formation of intracranial aneurysms. Drug candidates were set to target inflammatory processes for example statins, inhibitors of mast cell degranulation, TNF-α blockers, matrix metalloprotease inhibitors, free radical scavengers and tetracyclines[3]. While some of these animal studies were encouraging, only a limited number of drugs such as statins[4] (whose effect on aneurysm rupture are controversial[5,6]) and aspirin[7] continue to hold moderate promise in prevention of aneurysm rupture, though studies are limited.

With the emerging analytics in gene expression we are able to establish patterns for transcriptomics in bulk tissue[8–10] as well as on the level of cell population[11,12]. These technique yield relevant genetic signatures consisting of gene lists which can then be interpreted by linking to a unified nomenclature for biological processes in Gene Ontology[13,14]. The gene lists associated with a clinical phenotype can also be used to identify plausible drug candidates through the method of computational drug repurposing[15,16]. This technique relies on the assumption that if a drug which has opposing genetic signature to a disease, it may be a potential candidate for “reversing” the phenotype. This approach has yielded multiple plausible drug candidates verified in experimental models for lung cancer[17], inflammatory bowel disease[18], amyotrophic lateral sclerosis[19], and meningiomas[20].

We hypothesize that in the multifactorial mechanisms leading to aneurysm rupture there exists a “driver process” on a cell population level which associates with aneurysm formation. We further hypothesize that these gene signatures will yield plausible drug candidates which map well to population level gene expression.

## Methods

### Data preparation

Data for this study was collected from the open genetic repository Gene Expression Omnibus (GEO)[21]. We searched for studies using only the term “intracranial aneurysm”, specifying human studies with expression profiles available. We then searched the matching datasets for those which included microarray data on both ruptured and unruptured cerebral aneurysm domes, yielding two datasets[22,23] with a cumulative sample size of 33 aneurysms. The first study (GSE13353), compared whole genome expression profiles between ruptured and unruptured intracranial aneurysm walls which were frozen subsequent to operative clipping. The second (GSE15629) used a similar approach and also included samples of middle meningeal artery, though these were omitted from our analysis. The mean age of our merged cohort is 56.5, and the relative proportion of males to females is 36.4% to 63.6%, respectively. Further details of our study cohort can be found in Table 1.

**Table 1:** Summary of study population.

Series	Patients (N)	Ruptured (N)	Mean TTS* (SD)	Aneurysm Location (n)				
				MCA	ICA	AComm	PComm	PC
GSE13353	19	11	2.1 (3.4)	14	2	2	1	0
GSE15629	14	8	7.0 (10.5)	7	1	3	1	2
Total	33	19	4.3 (7.6)	21	3	5	2	2

*Time to surgery in days (ruptured aneurysms only). MCA = Middle Cerebral Artery, ICA = Internal Carotid Artery, AComm = Anterior Communicating Artery, PComm = Posterior Communicating Artery, PC = pericallosal artery

### Pre-processing of transcriptomics data

For each study, the microarray data was backgrounded corrected, quantile normalized, and log-2 transformed using the *Affy*[24] R package. Keeping only genes common to both studies (n = 18028), the datasets were merged, scaled to a global mean and standard deviation of 0 and 1, respectively[25], and batch-corrected using *ComBat*, a well-established empirical Bayes approach[26]. The resultant data matrix was used during all subsequent analysis.

### Co-expression network analysis

We used *Weighted Gene Co-Expression Network Analysis* (WGCNA) to identify biological “modules” (groups of biologically related genes) which map to aneurysm rupture. First, absolute Pearson coefficients were computed between each pair of genes, and the resultant matrix soft-thresholded with a hyperparameter in order to approximate scale-free topology (a desirable configuration for such network analyses). This was ultimately transformed into a biologically-inspired “Topological Overlap Matrix” (TOM) by expressing pairwise gene similarity in terms of their overlapping connectivity profiles, rather than direct expression correlations[27]. Highly similar genes were then grouped into an adaptive hierarchical clustering tree (dendrogram), yielding “modules” of co-expressed genes. The expression of each module is represented by a single meta-gene for each sample; using the first principal component is an established method to compute this value. A detailed description of this well-established methodology is described by Langfelder et al [8].

### Module-based qualitative analysis

Gene modules were queried using the Database for Annotation, Visualization and Integrated Discovery (DAVID version 6.8)[28], which assigns gene ontology terms to input gene lists. Gene ontology terms were considered significant if they achieved a Bonferroni-corrected p-values of <0.05.

### Computational drug repurposing

The technique of computational drug repurposing is founded on the basis that each disease state has a particular transcriptional “signature” of upregulated and downregulated genes. Likewise, exogenous perturbations to a biological system (i.e. the introduction of a drug) is associated with it’s own transcriptional signature. By cataloguing the signature of a variety of drugs using cell lines, one can match drug and disease signatures to select a compound which best acts to reverse a disease-specific signature of interest[16]. In this study, we used the state-of-the-art L1000 interface for drug repurposing [15]. To maintain a module-based approach, we first selected the modules which had significant positive correlation with aneurysms rupture (p<0.05). The top 10 genes in these modules (ranked by their correlation with module meta-gene expression), were input into the L1000 model as upregulated genes. This was repeated for modules negatively correlated with aneurysm rupture.

### Deconvolution of tumor bulk expression signal

We used *Population Specific Expression Analysis* (PSEA)[11], a well-established technique, to deconvolve cell-specific gene expression profiles from bulk data. This function uses a regression model to examine the transcriptomic contributions of particular cell types based on previously established marker genes, thereby mimicking single-cell data. The biological annotations of significant modules were used to select cell types of interest, and marker genes were selected from the literature (Table 2). A gene was considered to be significantly associated with a particular marker gene/genes (cell type) if it satisfied the following conditions in the regression model: a positive correlation with a p-value <0.05, an adjusted R^2^ >0.6, and a ratio of fitted intercept over mean expression < 0.5[11]. Output gene lists were investigated with DAVID, and gene ontology (GO) terms were compared between unruptured and ruptured aneurysms using the *GoSemSim* (“GO semantic similarity”) R package[29]. This flexible interface computes a similarity metric ranging from 0 to 1 between gene sets, GO terms, or sets of GO terms based (in oversimplified terms) on their proximity within a hierarchical graph depicting known biological associations and evolutionary relationships between genes or GO terms[29]. Mechanisms most specific to aneurysm rupture were of particular interest.

**Table 2:** Gene markers for each cell type used in population specific expression analysis.

Reference cell	Marker gene(s)
Smooth muscle[43]	MCAM, DES
Endothelial cell[44–47]	TJP1, SLC2A1, VCAM1, SELE, VWF
Macrophage[48–51]	CD14, FCGR1A, CD68, TFRC, CCR5
Mast cell[52]	KIT, ENPP3
T cell[53]	CD3D, CD3E, CD3G, CD4, CD8A, CD8B, IL2RA, IL7R, CCR6

### Computational platform

All analysis was done using the open-source platform for statistical computing R[30].

## Results

### Co-expression network and module analysis

**Fig 1:**
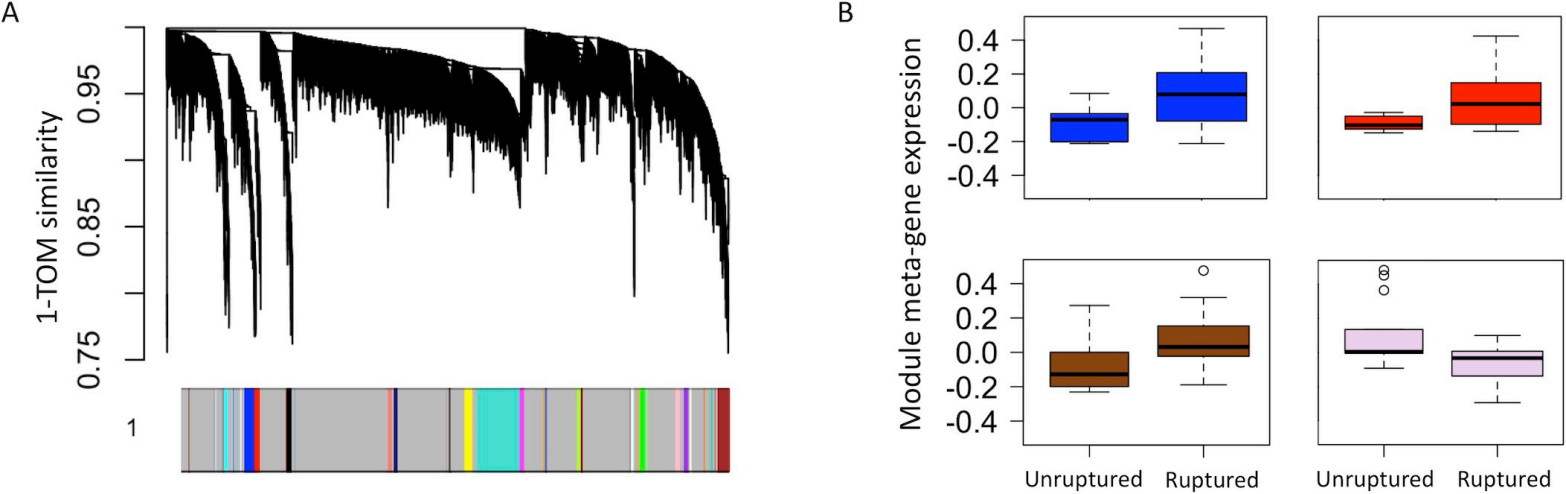
Module-based analysis reveals differences between immune processes in ruptured vs unruptured aneurysms. A: Dendrogram demonstrating the taxonomic relationship between genes with the y-axis representing gene dissimilarity based on the TOM metric. Colour bar representing genes grouped into modules. Grey interspacing represents unclassified genes. B: Boxplots of module meta-gene expression for significant modules which annotate to immune function. Both the blue and red modules (320 and 164 genes, respectively) annotate to inflammation/innate immunity. Similarly, the brown module (29 genes) annotates mostly strongly to Il-1 and TNF, while the lavender module (4 genes) enriches in B cells and phagocytosis predominantly. The horizontal lines represent median value, with the box representing interquartile range (first and third quartile represented by the bottom and top of the box, respectively). Whiskers represent the data range, with a maximum extension of 1.5 times the interquartile range. Values falling outside this range are considered outliers and represented by small circles.

Our approach yielded a total of 55 co-expression modules, ranging in size from 2 to 1381 genes (mean 322). The meta-gene expression of 12 modules differed significantly between ruptured and unruptured aneurysm cohorts (Mann Whitney p<0.05), indicating mechanisms associated with rupture (S1 Fig). Seven of these significant modules mapped significantly to various biological processes in DAVID (Bonferroni p<0.05), four of which were related to immune function (Fig 1).

### Computational drug repurposing

The L1000 software identified several compounds which may serve as potential drug candidates for aneurysm rupture. Each compound in the query is assigned a score, ranging from -100 to 100, which is a measure of how well it’s genetic signature matches the disease (a score of 100 is a perfect match, while a score of -100 indicates an effect that is exactly opposite to what is desired). In our analysis, 824 compounds were identified with positive scores, 164 of which had a score greater than 90 and 25 of which had a score >99, indicating extremely good match. The top 10 candidates are listed in Table 3.

**Table 3:** Module-based drug repurposing output reveals several potential drug candidates for aneurysm rupture. Top 10 compounds by score (a metric of how well the drug-disease signatures match) and their mechanisms are listed, with PKC activators, BTK inhibitor, and Nedd activating enzyme inhibitor being the highest ranked mechanisms.

Compound	Mechanism	Score
Prostratin	PKC activator	99.93
Terreic-acid	BTK inhibitor	99.93
Phorbol-12-myristate-13-acetate	PKC activator	99.93
Ingenol	PKC activator	99.93
MLN-4924	Nedd activating enzyme inhibitor	99.93
16,16-dimethylprostaglandin-e2	Prostanoid receptor agonist	99.89
VU-0418947-2	HIF modulator	99.86
Lypressin	Vasopressin receptor agonist	99.82
QW-BI-011	Histone lysine methyltransferase inhibitor	99.82
QS-11	ARFGAP inhibitor	99.82

### Population-specific gene expression

**Fig 2:**
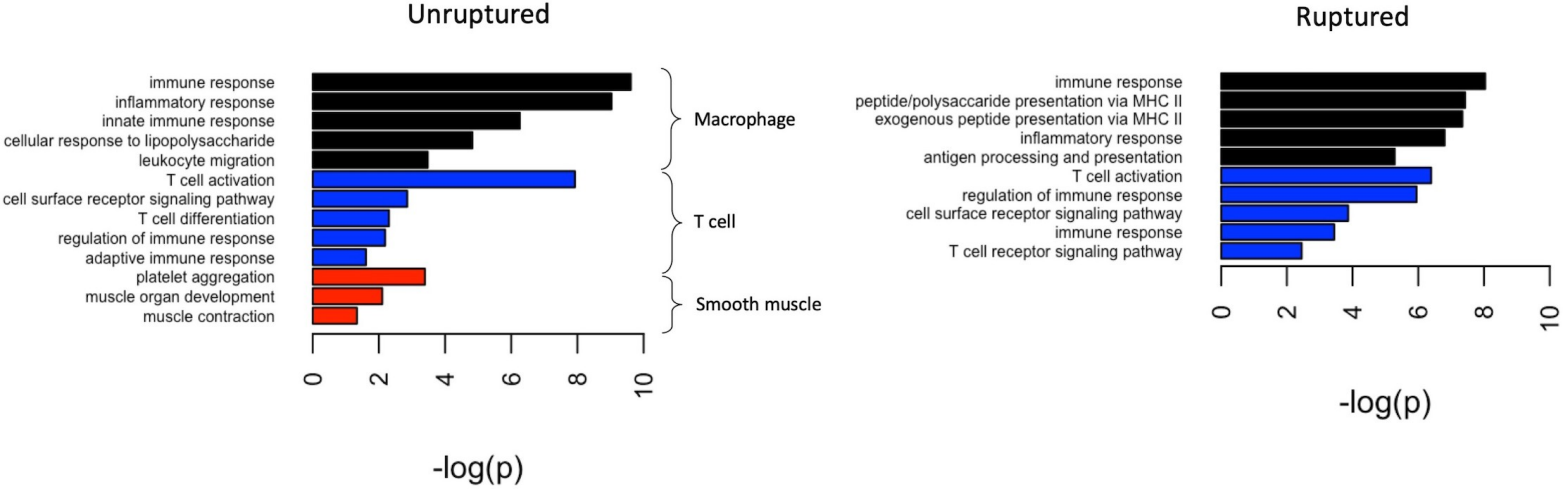
PSEA genes map to expected biological processes in DAVID. Barplots of the top 5 biological processes from each reference cell type, ranked by Bonferroni p-value. Endothelial and mast cell references did not yield any significant annotations in either cohort.

Cell-type specific expression for both ruptured and unruptured aneurysms was computed for smooth muscle cells, endothelial cells, mast cells, macrophages, and T cells using literature-derived marker genes (Table 2). For each of these 10 models, the genes which satisfied the criteria described in the *Methods* section were input into DAVID to investigate associated biological processes. Macrophage and T cell populations in both cohorts, as well as the smooth muscle population in the unruptured cohort, yielded significant enrichment while the other models did not (Fig 2). More specifically, “immune response” and “T cell activation” were the most significantly enriched gene ontology germs in both studies for macrophage and T-cell populations, respectively. In the macrophage population, the ruptured cohort had particular enrichment in processes relating to antigen presentation via MHCII and the unruptured cohort was enriched in innate immune processes including lipopolysaccharide response and leukocyte migration. The T-cell population enrichment terms were similar. Finally, “platelet activation” was the most significantly enrichment ontology term for the smooth muscle population in the unruptured population.

**Fig 3:**
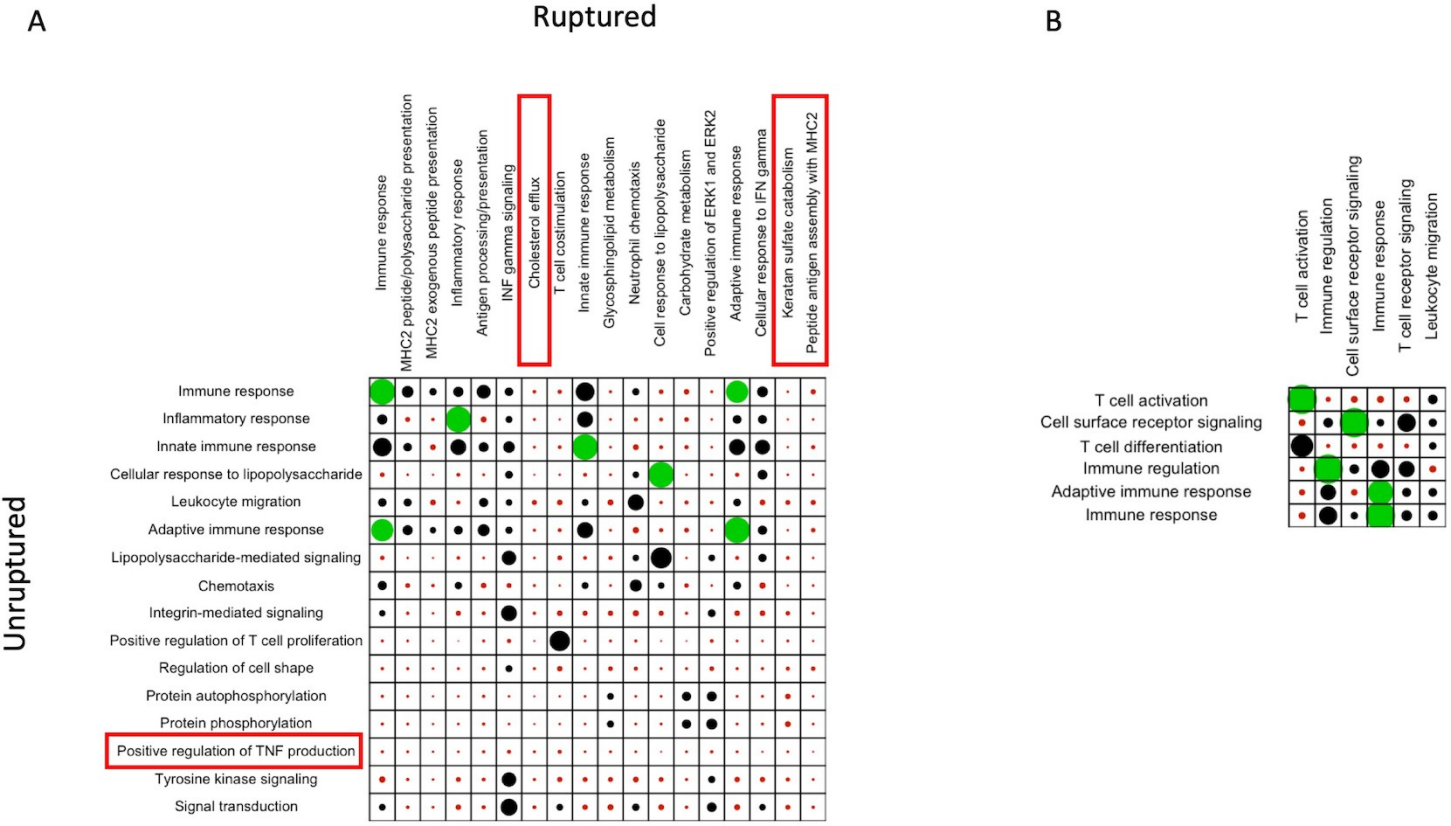
GoSemSim reveals differences in biological mechanisms between ruptured and unruptured aneurysms. A and B: Balloon plots of significant GO term similarity measure (S) for macrophage (A) and T cell (B) populations, with terms of ruptured and unruptured aneurysms represented as columns and rows, respectively. Dot size is proportional to S, and green dots represent high similarity (S > 0.8) while red dots represent low similarity (S<0.2). Red boxes highlight processes which have low association with the GO terms of the opposite phenotype (i.e. C_i_ for which S(A_i_,x)<0.2 for any x in D, where C and D represent phenotype).

To compare gene ontology outputs, the *GoSemSim* package was used to first assess the degree of overall similarity between gene sets generated from each of the 10 PSEA models (5 cell types). Subsequently, for cell types with enrichment in both unruptured and ruptured aneurysms (macrophages and T cells), the same package was used to compare significant individual ontology terms between cohorts (Fig 3). In the macrophage population, multiple processes were found to be particularly specific to ruptured aneurysms including antigen assembly with MHC2, keratan sulfate catabolism, and cholesterol efflux; positive regulation of TNF was more specific to unruptured aneurysms.

## Discussion

Our study is the first to apply system level genetic analysis to explore aneurysm microenvironment through transcriptomics data. It is also one of the few that directly compares unruptured and ruptured aneurysms[2], whereas many others compare to control vessel tissue (typically superficial temporal or middle meningeal arteries, which may not be representative of the vessels within the circle of Willis). We find multiple intuitive mechanisms enriched in the genetic signal from ruptured bulk aneurysm tissue strongly related to innate immune functions (Fig 1). Based on the genetic signatures of bulk aneurysm, we also identified multiple plausible drug candidates using computational drug repurposing, including Protein Kinase C (PKC) activators, Btk inhibitors, and HIF modulators (Table 3). We found that the effects of these drug candidates mapped well onto mechanisms related to antigen presentation, a process which was specific to the macrophage population within ruptured aneurysm wall. Finally, analysis of cell population-specific expression from macrophages and T cells using PSEA identified mechanisms specific to ruptured (antigen presentation, keratan catabolism, cholesterol efflux) and unruptured (TNF production) aneurysms; which may further improve our understanding of the pathophysiology of aneurysm rupture (Fig 3).

### Co-expression networks provide a novel perspective on aneurysm pathophysiology

Several previous studies have investigated the genetic basis of intracerebral aneurysm rupture using traditional differential gene expression, yielding hundreds to thousands of individual up- and down-regulated genes[2,31]. However, this model of isolated genes does not necessarily reflect biology, wherein genes have been found to better modeled in highly connected networks[27,32,33]. We therefore used WGCNA, a technique built to model genes closer to the way they exist in biological systems which separates them into domains (modules) of biological functions[27]. This approach is more likely to capture subtle effects from a large group of co-expressed genes which may be missed in single-gene analyses. We derived twelve modules whose meta-gene expression level differed significantly between ruptured and unruptured aneurysms (S1 Fig), seven of which mapped significantly to gene ontology processes in DAVID (Bonferroni p<0.05) with four relating to immune function (Fig 1). This is in keeping with previous work using single-gene analysis, which repeatedly suggests that the immune system plays a key role in aneurysm rupture[2,22,31,34]. In most of these studies, there appears to be an overall upregulation of immune function in ruptured tissue, which is consistent with our module-based findings.

### New drug candidates for aneurysm rupture from systems biology approach

Computational drug repurposing offers several benefits over the more traditional process of drug development, particularly due to the comparatively low cost, high efficiency, and data-driven nature. It has previously led to novel treatment candidates for diseases such as ALS[19], IBD[18], and cancer[17]. We identified several new drug candidates for aneurysm rupture, including Protein Kinase C (PKC) activators, Btk inhibitors, and HIF modulators, using representative module genes. PKC plays a role in several signal transduction pathways and can exist in various isoforms. It can play both a pro- and anti-inflammatory role in the immune system, and inhibitors of some of its specific isoforms have been trialed for use in diabetic microvascular disease and myocardial infarction[35]. Therefore, the role of PKC activators in intracerebral aneurysms is not immediately clear from the literature. The role of Btk, however, is better defined. Through interaction with Major Histocompatibility Complex (MHC) II, it ultimately triggers the release of pro-inflammatory cytokines from cells including macrophages[36]. Given the overexpression of multiple immune/inflammatory gene modules in aneurysm rupture, it is conceivable that blocking an important mediator of inflammation would reduce the risk of rupture. Similarly, the hypoxia-inducible transcription factor (HIF) is associated with macrophage activity and interferon gamma levels[37] and were previously found to be enriched in ruptured aneurysm tissue[34]; HIF modulators therefore make sense as a drug candidate as well based on its function.

### Population-specific analysis yields mechanisms specific to rupture

Population-specific gene expression has become an established method for analysis gene expression from histologically heterogeneous samples[11,12]. It relies on linear regression models to assess the fit of each gene from a bulk sample against a set of “marker” genes which represent a particular cell type. Genes whose expression pattern do not fulfill a set of criteria indicating their association with marker genes are filtered out, and remaining genes can be examined in terms of model coefficients[11]. This method has been used to demonstrate cell-specific expression changes in Huntington’s disease[11] and within the normal human cerebellum[12]. In our analysis, we probe the population-specific signals from smooth muscle, endothelium, mast cells, macrophages, and T cells to explore the GO mechanisms associated with these cell-specific gene lists. Macrophage and T cell-specific analysis yielded genes from both unruptured and ruptured phenotypes which enrich in several expected mechanisms (“immune response” and “T cell activation” being the most significant GO terms in both groups for each, respectively; Fig 2). Comparison of enriched mechanisms between both cohorts revealed processes associated with aneurysm rupture which were highly dissimilar with all processes of the unruptured cohort (S<0.2). In particular, keratan sulfate catabolism, cholesterol efflux, and antigen assembly via MHC II fulfilled this criterion and were therefore considered to be mechanisms specific to rupture in the macrophage-specific model, while TNF production was specific to unruptured aneurysms. Conversely, none of the enriched GO terms fulfilled this criterion in the T-cell-specific model (Fig 3).

The major histocompatibility complex (MHC) II plays a key role in immune function. It’s upregulation has previously been shown to associate with aneurysm formation and rupture[38], though we are the first to isolate this mechanism in a direct comparison of unruptured vs. ruptured aneurysms using macrophage-specific expression. Further, MHCII and other immune-related mechanisms are closely associated with Btk[36] and HIF[37] thus demonstrating overlap between our computational drug repurposing results, and the enriched mechanisms derived from population-specific gene expression. Finally, the role of cholesterol efflux less clear. It has previously been suggested that statins may prevent the formation and rupture of aneurysms[4], but this finding has been refuted by other studies which find no effect[5,6]. Interestingly, statins can have the effect of either increasing or decreasing macrophage-mediated cholesterol efflux, though with an overall preference for inhibiting this process[39]. This would lend theoretical mechanistic credibility to the potential utility of statins as a rupture-prevention therapy via the reduction of macrophage-associated cholesterol efflux (found to be associated with rupture in our results). Keratan sulfate (KS) is a complex glycosaminoglycan (GAG) with several structurally and functionally diverse proteoglycan forms including fibromodulin, which is associated with collagen production and inflammatory cytokines[40]. More generally, it has been shown that that total amount of GAGs is lower in aortic aneurysms compared to normal aortas[41]. It is therefore plausible that this represents another mechanism of aneurysm formation/rupture, thus corroborating our finding outlined above. Finally, tumour necrosis factor (TNF), the only mechanism specific to unruptured aneurysms, has been shown to associate with aneurysm pathogenesis and rupture [42]. We hypothesize that the lack of this signal in ruptured aneurysm is largely due to the signal being clouded by a number of other inflammatory mechanisms associated with the ruptured state, though this cannot be determined from our data.

### Limitations and future directions

There are multiple limitations of our study which must be considered. Firstly, the sample size is relatively small, which may affect the generalizability of results. Nevertheless, it is still larger than the majority of similar previous studies. Further, we did not have control over data collection as we used open repositories and therefore the data quality is not certain. We were nevertheless able to identify robust biological signal even in such heterogenous sample. Another inherent limitation is the inability to determine which effects occur subsequent to aneurysm rupture (rather than effects which predate rupture and therefore may serve as targets for rupture prevention). For example, immune activation may occur secondary to rupture.

Future work will be needed to confirm and expand upon our current results. Larger, prospective studies would be valuable to achieve greater generalizability and study control. Confirmatory testing of proposed mechanisms with immunohistochemistry would also be valuable. Finally, testing proposed drug candidates by analysis of electronic health records and by treating cell lines directly would be the next step in developing an effective medical therapy for the prevention of intracranial aneurysm rupture. Given the rarity of these medications, however, it is likely that the former would be challenging.

### Conclusion

This study is the first to use gene co-expression networks and population-specific transcriptomics to investigate the pathophysiology of intracerebral aneurysm rupture. We identified multiple mechanisms of rupture, which have logical footing based on previous literature, as well as several new drug candidates for the potential prevention of aneurysm rupture.

## Supporting information

S1 Fig Twelve modules were found to have significantly different average expression levels between unruptured and ruptured aneurysms (p<0.05). Seven of these map significantly to GO processes in DAVID (labeled). 0 = unruptured, 1 = ruptured.
(TIF)
