# Supplementary figures and images for: Microenvironment of ruptured cerebral aneurysms discovered using data driven analysis of gene expression
Source: PLoS One. 2019 Jul 22;14(7):e0220121. doi: 10.1371/journal.pone.0220121 (PMC6645676; doi:10.1371/journal.pone.0220121)

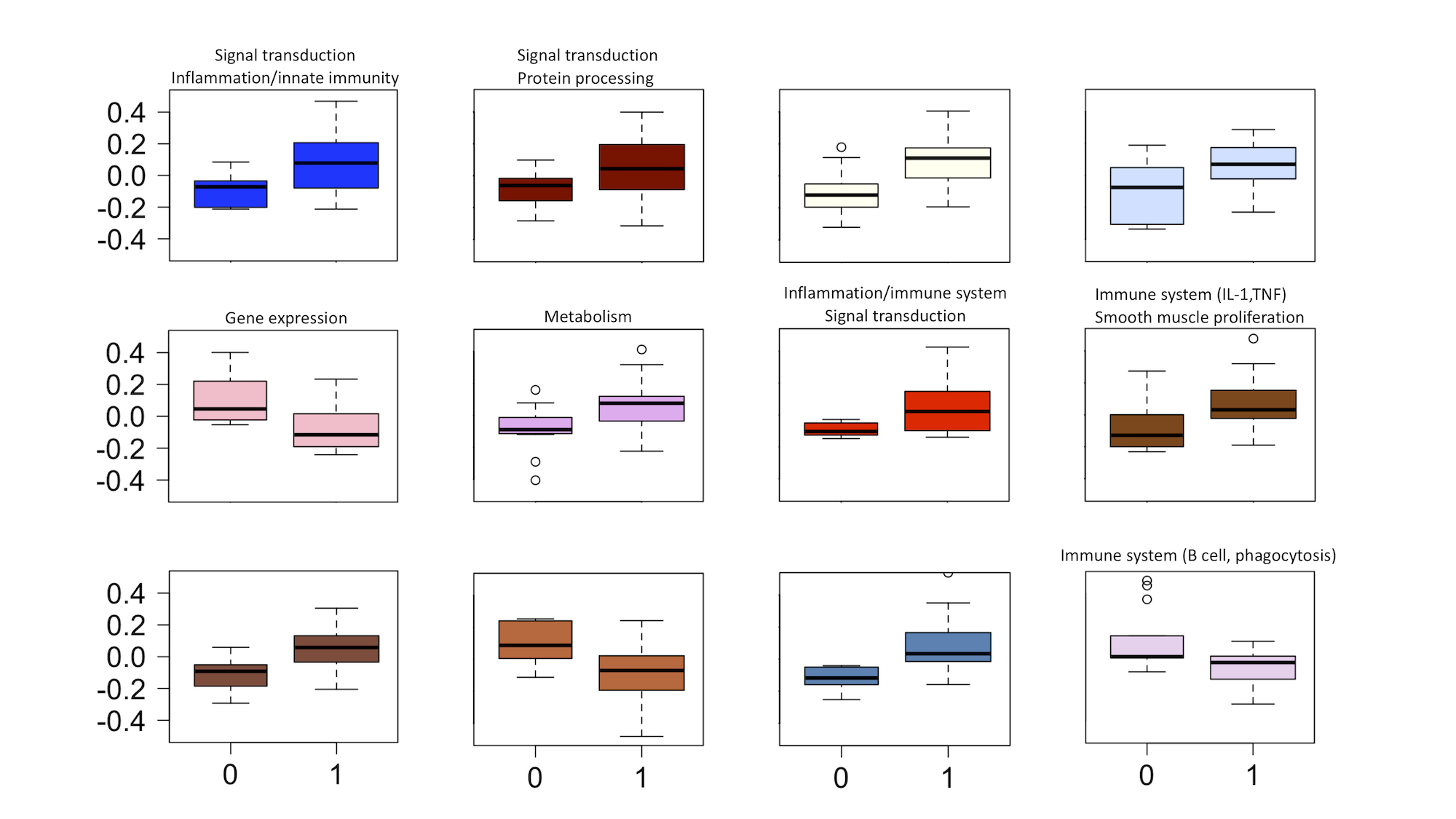

Supplement: S1 Fig — Seven of these map significantly to GO processes in DAVID (labeled). 0 = unruptured, 1 = ruptured. (TIF) [file pone.0220121.s001.tif]
